# Analysis of Whole-Genome facilitates rapid and precise identification of fungal species

**DOI:** 10.3389/fmicb.2024.1336143

**Published:** 2024-03-04

**Authors:** Guihong Qi, Lijun Hao, Tianyi Xin, Yutong Gan, Qian Lou, Wenjie Xu, Jingyuan Song

**Affiliations:** ^1^Key Lab of Chinese Medicine Resources Conservation, State Administration of Traditional Chinese Medicine of the People’s Republic of China, Institute of Medicinal Plant Development, Chinese Academy of Medical Sciences & Peking Union Medical College, Beijing, China; ^2^Engineering Research Center of Chinese Medicine Resource, Ministry of Education, Beijing, China

**Keywords:** Analysis of whole GEnome, AGE, fungal species identification, sequencing, genome editing

## Abstract

Fungal identification is a cornerstone of fungal research, yet traditional molecular methods struggle with rapid and accurate *onsite* identification, especially for closely related species. To tackle this challenge, we introduce a universal identification method called Analysis of whole GEnome (AGE). AGE includes two key steps: bioinformatics analysis and experimental practice. Bioinformatics analysis screens candidate target sequences named Targets within the genome of the fungal species and determines specific Targets by comparing them with the genomes of other species. Then, experimental practice using sequencing or non-sequencing technologies would confirm the results of bioinformatics analysis. Accordingly, AGE obtained more than 1,000,000 qualified Targets for each of the 13 fungal species within the phyla Ascomycota and Basidiomycota. Next, the sequencing and genome editing system validated the ultra-specific performance of the specific Targets; especially noteworthy is the first-time demonstration of the identification potential of sequences from unannotated genomic regions. Furthermore, by combining rapid isothermal amplification and phosphorothioate-modified primers with the option of an instrument-free visual fluorescence method, AGE can achieve qualitative species identification within 30 min using a single-tube test. More importantly, AGE holds significant potential for identifying closely related species and differentiating traditional Chinese medicines from their adulterants, especially in the precise detection of contaminants. In summary, AGE opens the door for the development of whole-genome-based fungal species identification while also providing guidance for its application in plant and animal kingdoms.

## 1 Introduction

Fungi support all life on Earth (Gonçalves et al., 2021); they are integral ecosystem agents that govern soil carbon cycling, plant nutrition, and pathology (Tedersoo et al., 2014). Thus, rapid and precise identification is crucial to understanding the fundamental influence of fungi and how they shape ecosystems (Peay et al., 2016). Fungi are extremely diverse and often lack discriminatory morphological characteristics (Kõljalg et al., 2016). Thus, for accurate species identification, molecular techniques may be necessary along with morphological traits. The DNA barcoding approach, which benefits from the rapid development of sequencing technologies, might currently be the best solution for identifying fungal species.

The nuclear ribosomal internal transcribed spacer (ITS) region, recognized as a universal DNA barcode for fungi, spans approximately 600 base pairs and comprises two variable spacers (ITS1 and ITS2), separated by the highly conserved 5.8S rRNA gene. ITS exhibits a well-defined barcode gap and significantly high PCR amplification success rates, enhancing its utility for species identification (Martinez et al., 2004; Schoch et al., 2012; Blaalid et al., 2013). However, a thorough study of ITS sequences in the International Nucleotide Sequence Database (INSD: GenBank, EMBL, and DDBJ) revealed that this region does not always exhibit sufficient variability to achieve differentiation between distinct species (Nilsson et al., 2008), particularly within the genera *Aspergillus* (Skouboe et al., 1999), *Cladosporium* (Schubert et al., 2007), *Penicillium* (Skouboe et al., 1999), and *Fusarium* (O'Donnell and Cigelnik, 1997). Thus, several other markers are employed, including translation elongation factor 1-α (TEF 1α) (Stielow et al., 2015), beta-tubulin gene (BenA), calmodulin (CAM), and RNA polymerase II gene (RPB2) (Raja et al., 2017). Although they often had a higher percentage of correct identification, low PCR amplification and sequencing success eliminated them as candidates for a universal barcode (Schoch et al., 2012). Therefore, developing new methods applicable to the entire fungal kingdom remains a major challenge.

Whole-genome sequencing offers the opportunity to facilitate extensive research on fungal taxa, accompanied by the rapid expansion of fungal species genomes. The number of fungal genome sequences available has grown exponentially: to date [5 November 2023, National Center for Biotechnology Information (NCBI)], approximately 16,646 whole-genome fungal sequences are publicly available. Simultaneously, the field of bioinformatics has undergone substantial growth, facilitating the analysis of extensive multi-dimensional datasets and yielding valuable biological insights. In addition, methods for quantifying fungal diversity have evolved substantially in recent decades. High-throughput sequencing (HTS), shotgun sequencing, and targeted metagenomics have unveiled a wealth of previously undiscovered biodiversity (Lofgren and Stajich, 2021). The increasing availability of genomic sequences has contributed to the construction of the Universal Fungal Core Genes (UFCG) database and pipeline for genome-wide phylogenetic analysis of fungi (Kim et al., 2023). Core gene-based automated phylogenomic pipelines, like the Genome Taxonomy Database (Parks et al., 2022) (GTDB), AutoMLST (Alanjary et al., 2019), and UBCG (Kim et al., 2021), are now widely adopted in the field.

**Figure 1:**
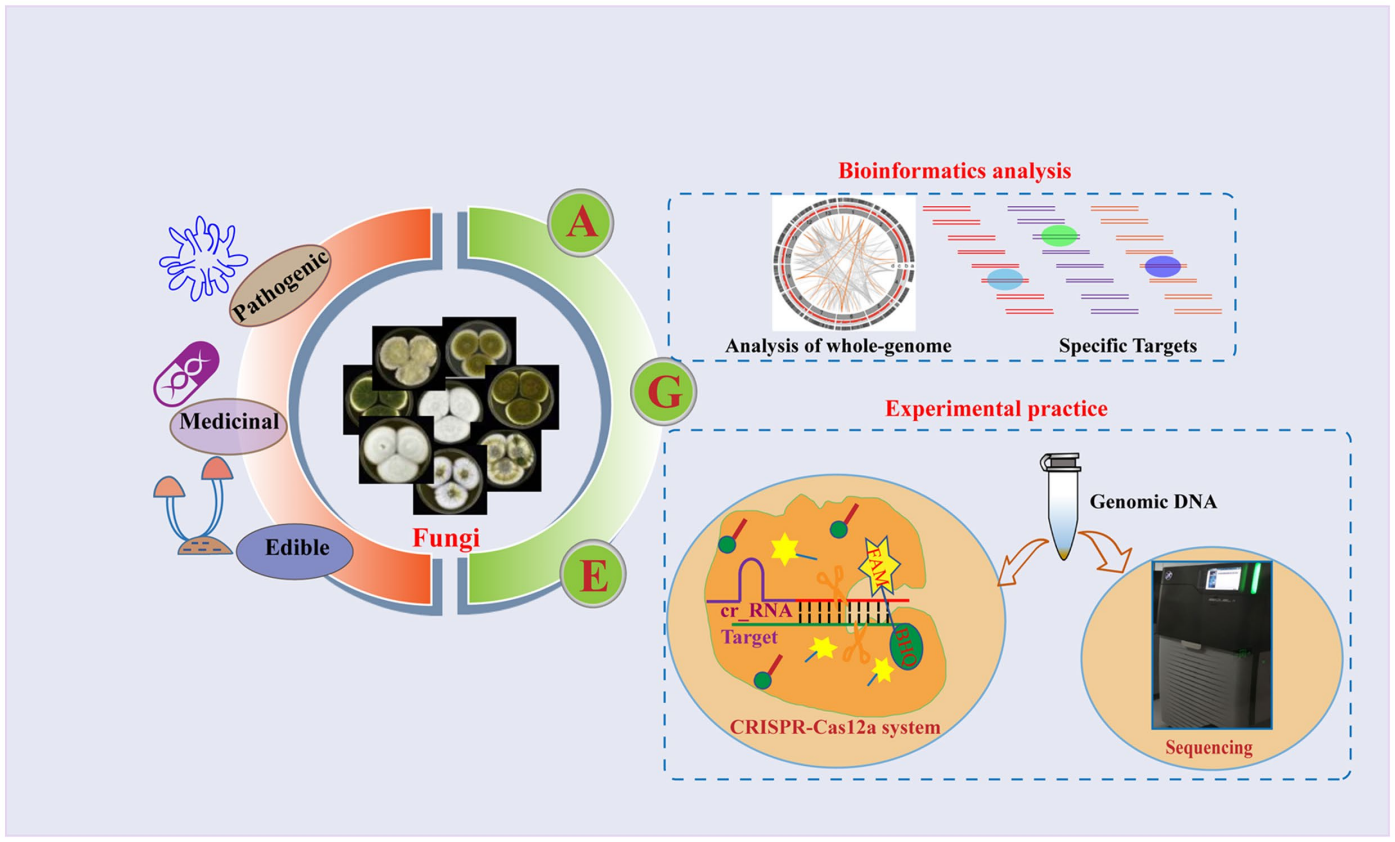
Overview of the AGE assay. Fungi are among the most diverse organisms on the planet and play a crucial role in ecosystem processes and functioning (Hyde, 2022). Some species of fungi can cause severe fungal infections that pose a threat to human health, while others have a history of being used as food or medicine for centuries. Due to the complex diversity of fungi, accurate identification of fungal species has become particularly important. As a novel approach to species identification, the basic principle of AGE relies on the fact that different species have distinct whole genomes. It is a methodology consisting of two steps: bioinformatics analysis and experimental practice. Using bioinformatic analysis, the candidate target sequences named Targets are screened from the whole genome, and the specific Targets are identified by comparison with the genomes of other species. To accomplish the specific Target recognition, the specific Target sequences named Target DNA are first enriched in genomic DNA using specific primer pairs. Different techniques were then utilized to recognize the specific Targets, including sequencing and the CRISPR-Cas12a system. In the sequencing, the amplified products were sequenced, and the identity of the sample with the target sequences was determined by alignment. In the CRISPR-Cas12a system, crRNA corresponding to the Target is synthesized, and the Cas12a protein is incubated with it to form the Cas12a-crRNA complex. Subsequently, the Target DNA and the single-stranded DNA (ssDNA) reporter were coupled with the Cas12a-crRNA complex. The ssDNA contained a 6-carboxyfluorescein (FAM) reporter dye at the 5′-end and a Black Hole Quencher (BHQ) at the 3′-end. Finally, the fluorescence signals are generated *via* ssDNA breaks and recorded by a fluorescence microplate analyzer. The morphological characteristics of the various species depicted in the figure were cited by Frisvad et al. (2019).

To achieve universal fungal species identification, we present a novel strategy for fungal identification based on the Analysis of whole-GEnome (AGE), with the support of bioinformatics analysis and technique development. AGE is capable of identifying species through rigorous bioinformatics analysis and employs various techniques for specific target detection. It is a methodology consisting of two stages: bioinformatics analysis serves as the initial step, responsible for generating Target library within species genomes and filtering for species-specific Targets through comparisons with the genomes of other species. Subsequently, the genomic DNA of the species is extracted, and the sequencing or CRISPR-Cas12a system is employed to detect the selected specific Targets (Figure 1). For the Cas12a system, a single CRISPR RNA (crRNA) with a T-rich protospacer adjacent motif (PAM) sequence serves as a guide to bind and cleave corresponding double-stranded DNA (dsDNA). After being target-activated, Cas12a shows the ability to cleave non-specific single-stranded DNA (ssDNA) (Chen et al., 2018). Recent studies have shown that the CRISPR-Cas12a systems can actualize the rapid detection of viruses (Chen et al., 2018; Kellner et al., 2019), fungi (Lei et al., 2022; Mu et al., 2022), and bacteria (Liu et al., 2021), paving the way for their applications to species identification of fungi.

In our study, we chose *Ganoderma lucidum* as a model species to construct the identification platform since it was an economically and culturally significant medicinal mushroom that had been used in East Asian countries for over 2,000 years (Pan et al., 2019; Wang et al., 2019) and had completed fine-mapping at the chromosome level (Chen et al., 2012). Additionally, based on four considerations—each fungal species must (i) have its own genome, (ii) be from a different class, (iii) have different functional traits, and (iv) be easily available—we included 12 additional fungal species within the phyla Ascomycota and Basidiomycota in our testing to showcase the versatility of AGE and gain further insight into its potential. *Aspergillus flavus* is a major cause of severe non-invasive fungal infections in Middle Eastern countries, and it is difficult to distinguish from *Aspergillus oryzae* (Hedayati et al., 2019), making it a good example to illustrate the application of AGE in identifying closely related species. All results showed AGE is a simple, fast, and reliable method that will aid in addressing many key challenges in fungal identification. If implemented on a larger scale, this method has the potential to become a staple in the identification process of many other species outside of the fungal world.

## 2 Materials and methods

### 2.1 Materials preparation

Thirteen species were chosen to be investigated using the AGE method. The standard strains of *Alternaria alternata* (Fr.) Keissl, *Agaricus bisporus* (J.E. Lange) Imbach, *Aspergillus flavus* Link, *Apiotrichum laibachii* (Windisch) A.M. Yurkov & Boekhout, *Botrytis cinerea Pers*, *Fusarium oxysporum Schltdl*, *Ganoderma lucidum* (Curtis) P. Karst, *Saccharomyces cerevisiae* Meyen ex E.C. Hansen, and *Rhodotorula mucilaginosa* (A. Jörg.) F.C. Harrison were obtained from the China General Microbiological Culture Collection Center (CGMCC, Beijing, China). *Auricularia heimuer* (Klotzsch) Mont, *Lentinula edodes* (Berk.) Pegler, *Ophiocordyceps sinensis* (Berk.) G.H. Sung, J.M. Sung, Hywel-Jones & Spatafora, and *Wolfiporia cocos* (Schwein.) Ryvarden & Gilb were purchased from the market. We also chose *Aspergillus oryzae Anderson* (a closely related species of *A. flavus*) obtained from CGMCC and *Cordyceps militaris* (L.) Link (the adulterant species of *O. sinensis*) purchased from the market to validate the accuracy of AGE. *A. alternata*, *A. laibachii*, *A. flavus*, *A. oryzae*, *B. cinerea*, *F. oxysporum*, *G. lucidum*, *R. mucilaginosa*, and *S. cerevisiae* are standard strains obtained from CGMCC and have undergone morphological identification. *A. bisporus*, *A. heimuer*, *L. edodes*, and *O. sinensis* were identified by morphological expert Lin Yu Lin. Detailed information is provided in Supplementary Table S4.

### 2.2 Bioinformatics analysis for the specific Target screening

The genomes of all analyzed species were downloaded from the NCBI database^1^; genome reference versions are listed in Supplementary Table S1. The genomes of these species were cut into 25-bp fragments using Jellyfish (v1.1.12) to generate (L-25 + 1) 25-mers with the copy number using the default parameters (L = genome length). The 25-mers with PAM sequences (TTTV starting, where V = G or C or A) were extracted and compared to their own genomes with Bowtie (v1.1.0) using the default parameters to obtain their locations in their corresponding genomes. All selected 25-mers were considered as candidate target sequences (Targets), and the crRNAs were designed based on the selected Targets according to the references (Zetsche et al., 2015; Moreno-Mateos et al., 2017).

As ITS has been the universal DNA barcode for fungal species identification (Schoch et al., 2012), we prioritize the analysis of Targets located in this region. For *G. lucidum*, the specificity of these Targets was determined by mapping the target sequences to the genomes of the 13 selected species using Cas-OFFinder (v2.4). From this, the off-Targets with PAM TTTV within five mismatches of the Target were obtained. For *O. sinensis*, the predicted off-Targets were achieved through aligning target sequences to the genome of *Cordyceps militaris*, which was the adulterant species of *O. sinensis*. Given that *A. flavus* did not have any specific target in the ITS region, we analyzed Targets located in other regions and obtained specific Targets by mapping these sequences to the whole genome of *A. oryzae*, a closely related species to *A. flavus*. Based on the selected specific Target, we predicted potential off-targets with up to five mismatches by aligning the target sequence with the genomes of other species. The corresponding crRNAs for specific Targets of each fungal species were synthesized (GenScript Co. Ltd., China).

### 2.3 Experimental practice for the specific Target recognition

#### 2.3.1 Sequencing for the Target recognition

##### 2.3.1.1 DNA extraction

The samples of *A. alternata*, *A. laibachii*, *A. flavus*, *A. oryzae*, *B. cinerea*, *F. oxysporum*, *G. lucidum*, *R. mucilaginosa*, *S. cerevisiae*, and *W. cocos* were grown on PDA medium, and an appropriate amount of mycelium was finely ground in liquid nitrogen, and DNA extraction was performed using a commercial genomic DNA extraction kit [DP305, Tiangen Biotech (Beijing) Co. Ltd., China] according to the manufacturer’s protocols. Then, the DNA quality and quantity were evaluated using a NanoDrop™ 2000 spectrophotometer (Thermo Fisher Scientific, USA) and 0.8% agarose gel electrophoresis in 1× TAE buffer at 140 V for 40 min (Bio-Rad Laboratories Inc., USA).

##### 2.3.1.2 Specific Target amplification and purification

For each species, we designed specific primers based on the 500-bp length upstream and downstream of the target and amplified the target using these primers. Except for *A. flavus*, the Targets are located in the ITS region, so the universal primer pairs can be used for their amplification. For *A. flavus*, the specific primer pairs were designed with the NCBI Primer-BLAST^2^. The genomic DNA of each species, flanked with primers listed in Supplementary Table S2, was used as a template for PCR amplification. The primers used were synthesized by GenScript Co. Ltd., China. PCR amplification of the Targets was performed in 25-μl reaction mixtures containing 30 ng of genomic DNA, 12.5 μL of 2× Taq PCR MasterMix (Aidlab Biotechnologies Co. Ltd., China), and 1 μL of each forward and reverse primers (2.5 μmol/L). Samples were amplified in an Applied Biosystems Veriti™ Thermal Cycler (Thermo Fisher Scientific, USA), and the reaction conditions were as follows: 5 min at 94°C, followed by 30 cycles of 1 min at 94°C, 1 min at 50°C, and 1.5 min + 3 s/cycle at 72°C, with a final step of 7 min at 72°C. The amplified DNA was purified according to the instructions of the QIAquick PCR Purification Kit (Qiagen Co. Ltd., Germany) and named the product *Gl*_target.

##### 2.3.1.3 Sequencing for the specific Target validation

The purified PCR products of each species were sequenced bidirectionally using Sanger sequencing. Contig assembly and the generation of consensus sequences were performed using the Codon Code Aligner. Low-quality sequence data and primer sequences were removed. The BLAST program from the NCBI website was used for sequence alignment.

#### 2.3.2 Non-sequencing system to recognize the specific Targets

##### 2.3.2.1 DNA extraction

Genomic DNA was extracted as above.

##### 2.3.2.2 Specific Target amplification and purification

Specific Target amplification and purification were performed as above.

##### 2.3.2.3 CRISPR Cas12a system for specific Target detection

CRISPR-Cas12a technology was employed as an exemplary approach to developing a non-sequencing system. *Ganoderma lucidum* was selected as the model species to construct the AGE identification platform. First, we examined the specificity of the *G. lucidum* crRNA. 50-μl 1× Cas12a reaction mixture containing 20-nmol/L Cas12a (New England Biolabs Co. Ltd., USA) and 40-nmol/L crRNA of *G. lucidum* (*Gl*_crRNA, GenScript Co. Ltd., China) was incubated at 37°C for 15 min to form the Cas12a-crRNA complex. The 5 μl of amplicons (30 ng/μl) of *G. lucidum* from the previous step was mixed with the Cas12a-crRNA complex reaction and incubated at 37°C for 1 h. The 5 μl of *Gl*_target from the previous step was mixed with the Cas12a-crRNA complex reaction and incubated at 37°C for 1 h. Then we incubated the mixture at 65°C for 10 min to stop the reaction and checked the cleavage of *Gl*_target by 2% (w/v) agarose gel electrophoresis in 1× TAE buffer at 120 V for 30 min.

Following this, the Cas12a-crRNA complex solution was prepared. It contained 10-μl NEBuffer 2.1 (10×), 2-μl Cas12a (1 μmol/L), 3.3-μl *Gl*_crRNA (10 μmol/L), and 64-μl nuclease-free water. Next, the solution was incubated at 37°C for 15 min (Bonini et al., 2021). Upon complex formation, 16.7-μl *Gl*_target (30 ng/μL) from PCR amplification and 4-μl ssDNA-A (10 μmol/L) were immediately added to it, and subsequently, fluorescence intensity was recorded by a microplate reader. The *Gl*_target from ERA amplification was not purified, and we directly added different amounts of it while changing the volume of nuclease-free water to keep the total volume (100 μl) constant. This final reaction mixture was incubated at 37°C and tested at 0, 3, 6, 9, 12, 15, 25, 35, 45, and 60 min. The fluorescence intensity was read at λ_ex_ 483 nm/λ_em_ 535 nm by the fluorescence microplate analyzer (Thermo Fisher Scientific, USA) at each time point.

#### 2.3.3 One-tube system for on-site Target recognition

Genomic DNA was extracted as above. Recently, the popularity of recombinase polymerase amplification (RPA) has enabled isothermal amplification of genomic DNA (Lobato and O'Sullivan, 2018). Enzymatic recombinase amplification (ERA), an isothermal PCR alternative based on RPA, was used to achieve room-temperature amplification. A volume of 3 μl of genomic DNA (10 ng/μl) was added to the reaction mixture, and amplification was performed according to the instructions of the ERA kit (KS101, Gendx, Co. Ltd., China). For this experiment, three different primer pairs (Supplementary Table S2) were selected to compare the amplification efficiency and visualize the results by 2% (w/v) agarose gel electrophoresis in 1× TAE buffer at 120 V for 30 min.

The ERA amplification reagents were added to the bottom of a 1.5-ml centrifuge tube. The Cas12a-crRNA complex was carefully added to the inner cap, including 2-μl Cas12a, 3.3-μl crRNA, 10-μl NEBuffer 2.1, and 30.7-μl nuclease-free water. After the amplification at 40°C for 20 min, the reaction was centrifuged briefly to draw the solution from the cap to the base of the tube, and the mixture was incubated at 37°C for 15 min. Then, 4-μl ssDNA was added to the mixture and tested for fluorescence signals as described above. In addition, a fluorescence microplate analyzer and visual fluorescence can also be used for the results readout. For visual fluorescence, 4-μl ssDNA-C was added to the mixture and incubated again at 37°C for 5 min. The mixture was subsequently observed by excitation using a wavelength of 470 nm. The quantification of the fluorescence images was performed using ImageJ software.

### 2.4 Statistical analyses

*P*-values were calculated using the one-way ANOVA (multiple groups). Data were expressed as mean ± SD. Differences with *p*-values <0.05 were considered significant. All statistical analysis was performed using the GraphPad Prism 8.0 software.

## 3 Results

### 3.1 The specific Target library of the fungal species

As a model species of medicinal fungi, genome sequence analysis revealed that there were 745,190 available Targets in *G. lucidum*, both with annotated and unannotated sequences. On average, there was one potential target sequence per 64 bp in the genome of *G. lucidum* (Supplementary Table S3). Subsequently, we analyzed the genomes of other fungal species that had been extensively studied, including closely related species, edible fungi, and traditional Chinese medicine. The Target numbers of these fungi ranged from 298,019 to 3,432,386, and the average distance between two Targets of different species varied greatly, such as 21 bp in *S. cerevisiae* and 90 bp in *A. laibachii* (Supplementary Table S3). Interestingly, all species except for *Aspergillus flavus* have found species-specific Target in the annotated ITS region, which can be utilized for subsequent experimental validation (Table 1). Among them, to assess the suitability of the specific Targets distinguishing *O. sinensis* from its adulterant *C. militaris*, we analyzed eight potential Targets in the ITS region of *O. sinensis*. The target *Os*_target 7, which has no off-target within five mismatches in *C. militaris*, was selected for experimental practice (Supplementary Table S4).

**Table 1:** Specific Targets for experimental practice on 13 fungal species.

Species	Specific Target sequence (5′ → 3′)
Agaricus bisporus*	TTTCAGAGGAGCTGACCCCAAGTAA
*Alternaria alternata*	TTTGCTGATAGAGAGTGCGACTTGT
*Apiotrichum laibachii*	TTTGAACGCAACTTGCGCTCTCTGG
*Aspergillus flavus*	TTTCGGAGTTCACCGGCATCAGTGC
Auricularia heimuer*	TTTCAAGACGAGCCGATTACCGGCA
*Botrytis cinerea*	TTTAGAGCCTGCCATTACTGACATA
*Fusarium oxysporum*	TTTGCTGCGTTCTTCATCGATGCCA
Ganoderma lucidum*	TTTGTAGGCTTGGACTTGGAGGCTT
Lentinula edodes*	TTTCTCCAATGAATAGAACAGATTGA
Ophiocordyceps sinensis*	TTTGGGAGTGGTGAC TCGATAATGA
*Rhodotorula mucilaginosa*	TTTACGGTCTAGCTCGTTCGTAATG
*Saccharomyces cerevisiae*	TTTAAGAACATTGTTCGCCTAGACG
Wolfiporia cocos*	TTTCTAGGGTTCCCGTTCAACGGCG

^*^The medicinal or edible species.

For *A. flavus*, the whole-genome analysis revealed that there were 1,073,076 Targets in it. On average, there was one potential Target sequence per 36 bp in the genome of *A. flavus*. Comparing the Targets of *A. flavus* to the whole genome of *A. oryzae*, a high degree of sequence similarity was observed at 92.2%. The Targets, particularly those located in the DNA barcode region such as ITS, BenA, and RPB2, are not specific. To demonstrate that each specific Target can be used in the subsequent Target recognition process, we selected ‘dark matter’ (an unannotated sequence within the genome) as the specific Target for *A. flavus*. Thus, we randomly selected a novel, previously unannotated sequence located on chromosome 6 of the *A. flavus* genome, referred to as *Af*_target (Table 1).

### 3.2 Sequencing successfully achieved species identification

**Figure 2:**
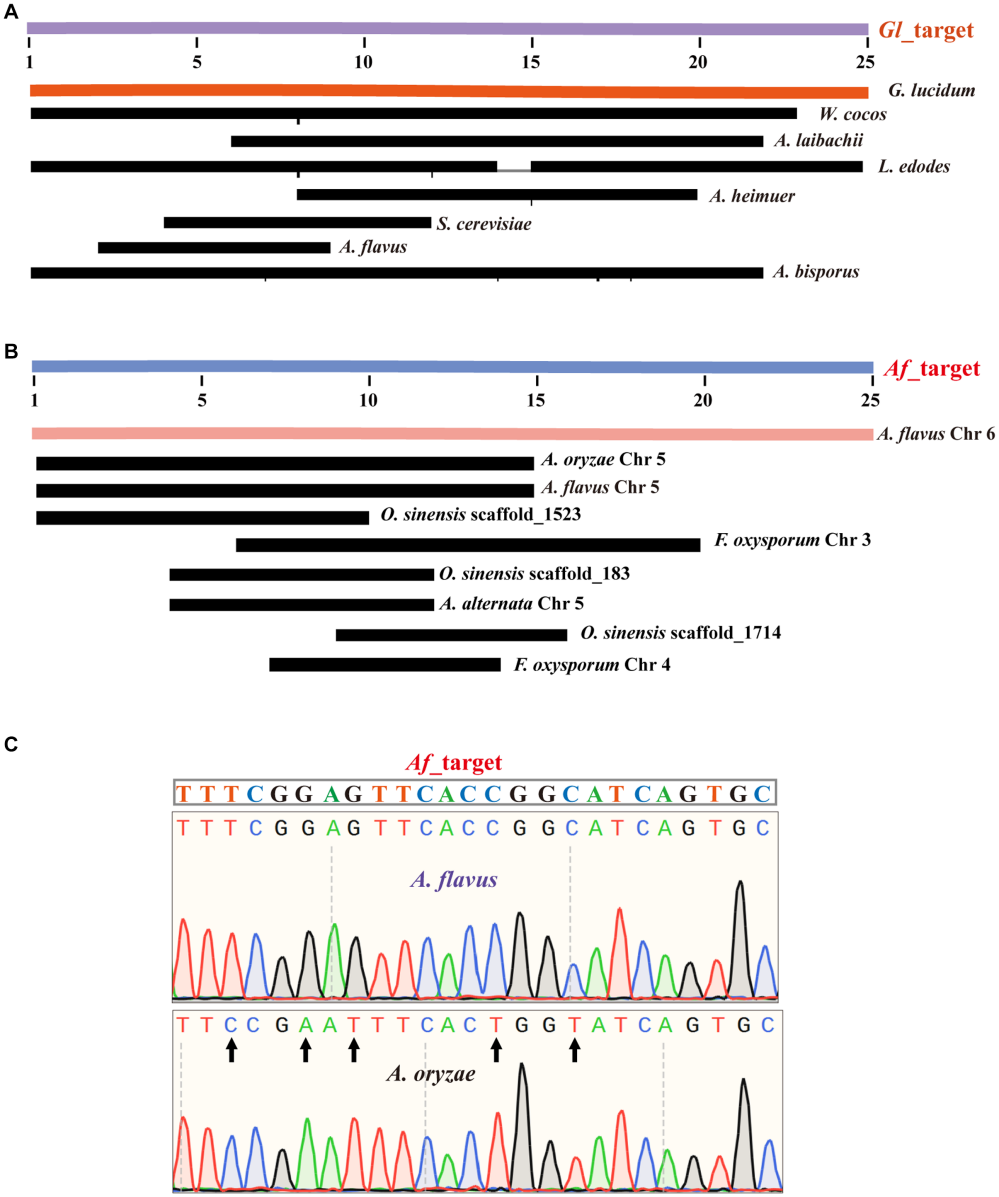
Successful sequencing achieved precise identification of Target sequences. **(A)** The alignment of the *Gl*_target sequence with ITS sequences from 13 species. **(B)** The alignment of the *Af*_target sequences with genomes from 13 species. **(C)** The alignment of the *Af*_target sequence with the amplicons from the *A. flavus* sample and the *A. oryzae* sample.

First, we amplified the ITS sequences of each species using universal primers (Supplementary Table S2). Subsequently, we performed a preliminary analysis of the function of *G. lucidum*’s specific Target (*Gl*_target). By comparing *Gl*_target with the ITS sequences of these 13 species, we found that only *G. lucidum*’s ITS sequence contained a sequence identical to *Gl*_target, while there were varying degrees of differences between the *Gl*_target sequence and the ITS sequences of other species. This difference supports *Gl*_target, which enables accurate identification of *G. lucidum* (Figure 2A). For the selected other fungi (except *A. flavus*), sequencing results demonstrated that their respective specific target sequences were able to achieve differentiation from other species (Supplementary Figure S1).

For *A. flavus*, our results are consistent with the known literature, which has exactly the same ITS sequence as *A. oryzae* (Supplementary Figure S2). Therefore, we randomly selected a specific target sequence for experimental validation and named it as *Af*_target. We first analyzed the genomes of 13 species for similarity sequences with five mismatches to the *Af*_target. The results showed that only *A. flavus* contained a sequence identical to the target. Furthermore, none of the other species had any similarity sequences with three mismatches. For similarity sequences with four or five mismatches, 1–3 sequences were present in some individual species (Figure 2B). In the upcoming experimental validation, we first designed one novel primer pair (Supplementary Table S2) to amplify the selected target sequence, and the specificity of amplification was verified by agarose gel electrophoresis (Supplementary Figure S3). Subsequently, we obtained the specific information of the amplified fragments by sequencing the amplified products. The sequencing results and bioinformatics analysis were consistent, indicating that *Af_target* can indeed serve as a specific target for distinguishing *A. flavus* from *A. oryzae*. For the *A. flavus* sample, we successfully identified a sequence that matched the *Af*_target completely in the sequencing results, while there were five base differences in the *A. oryzae* amplicons (Figure 2C).

### 3.3 Non-sequencing system enables highly specific, sensitive, and stable *Ganoderma lucidum* identification

**Figure 3:**
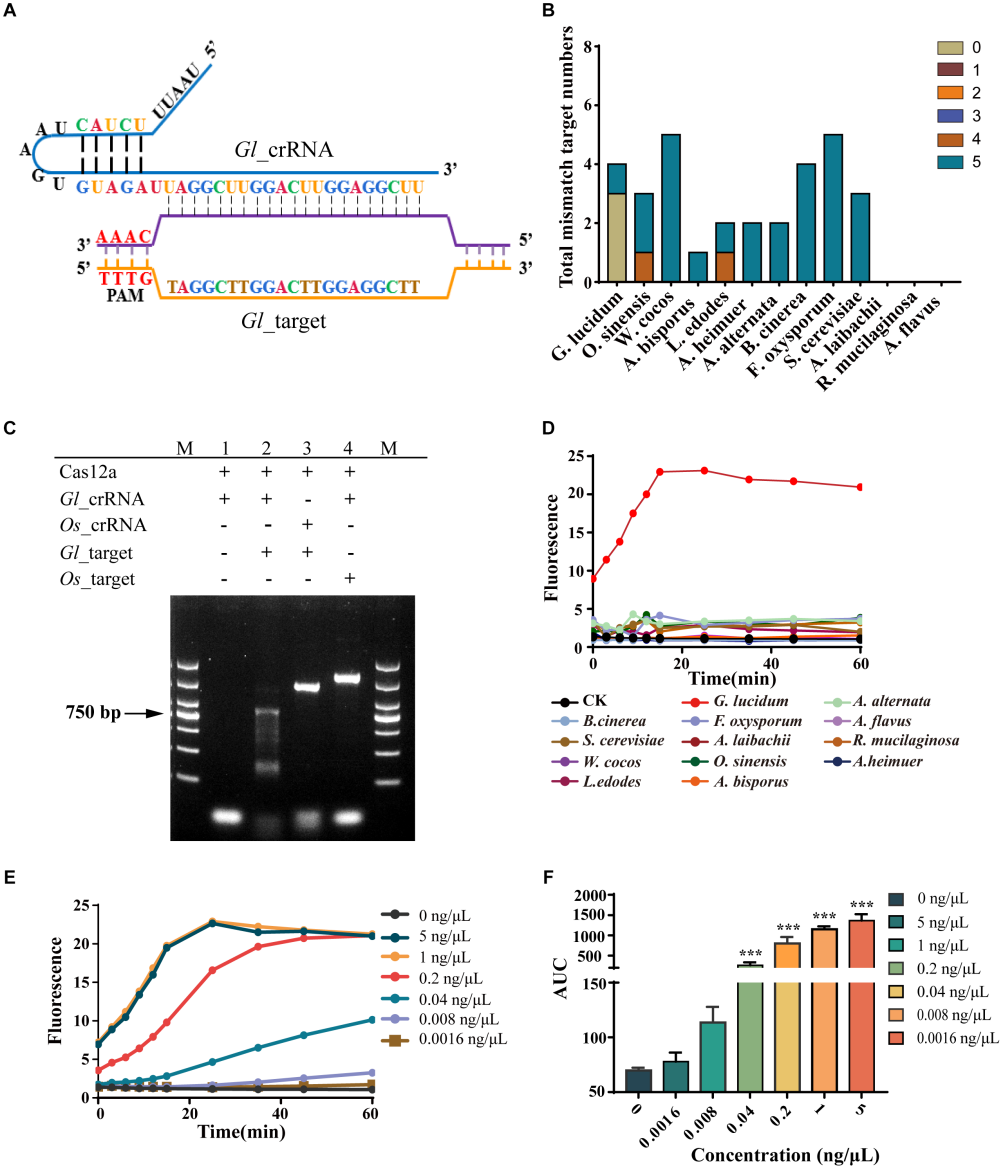
Non-sequencing system achieved highly specific, sensitive, and stable *G. lucidum* identification. **(A)** Binding and structure of *Gl_*target and *Gl*_crRNA. **(B)** The specific analysis of *Gl*_target. The target numbers with different mismatches of *Gl*_target in all 13 fungal species are examined by bioinformatics analysis. **(C)** Specificity of *Gl*_crRNA. The *Gl*_target is the ITS amplicons of *G. lucidum*, and the *Os*_target is the ITS amplicons of *O. sinensis*. M is the DL2000 DNA marker. (1) Cas12a + *Gl*_crRNA. (2) Cas12a + *Gl*_crRNA + *Gl*_target. (3) Cas12a + *Os*_crRNA + *Gl*_target. (4) Cas12a + *Gl*_crRNA + *Os*_target. **(D)** The specificity of AGE over time. CK group contains all reagents except the DNA substrate. *G. lucidum* group contains 1-ng/μl purified ITS amplicons of *G. lucidum*. The other group contains all the reagents and corresponding 1-ng/μl purified ITS amplicons for each species. **(E)** The concentration sensitivity of AGE by fluorescence over time. **(F)** The repeatability of AGE by concentration. Averaged results are reported as means of the area under the curve (AUC) of fluorescence. Error bars represent the mean ± SD, where *n* = 3. ***p < 0.001.

We designed the matching cr_RNA for *G. lucidum* according to the *Gl*_target, which we named *Gl*_crRNA (Figure 3A). Further specificity analysis by mapping the *Gl*_target to the genomes of other species in our study revealed that the target is unique to *G. lucidum*. In addition, we further analyzed the sequences with high similarity to the target sequences in each species and searched the whole genome of each species by setting different mismatch numbers. In *G. lucidum*, no similar sequences were found within three mismatches, and only one similar sequence was found when the number of mismatches reached five. The specificity of *Gl*_target was also demonstrated by the absence of similar sequences within two mismatches in other species, and only *O. sinensis* and *L. edodes* have one sequence with three mismatches, respectively. *A. alternata*, *A. bisporus*, *A. heimuer*, *B. cinerea*, *F. oxysporum*, *S. cerevisiae*, and *W. cocos* have several sequences with five mismatches. *A. laibachii*, *A. flavus*, and *R. mucilaginosa* have no sequence within five mismatches (Figure 3B).

As a tool for identifying Targets, we first examined the requirements for Cas12a to function. The result showed that Cas12a can only cleave the dsDNA when coupled with *Gl*_crRNA and *Gl*_target, which suggests that Cas12a can only be activated when both crRNA and the corresponding DNA are present. Considering *O. sinensis* has one sequence with three mismatches and *Gl*_crRNA combined, it cannot activate the Cas12a function, suggesting three mismatches are sufficient for on-target recognition. In addition, the efficiency of *Gl*_crRNA also indicated that the principle for Target screening is feasible enough (Figure 3C). As a novel identification method, AGE was validated for its specificity, sensitivity, and stability. First, we assessed the recognition capability of the *Gl*_crRNA in purified ITS amplicons across all fungal species, and the outcomes indicated that the *Gl*_crRNA is specific to the identification of *G. lucidum* species, as evidenced by significant fluorescent signals detected solely in *G. lucidum* samples via fluorescence microplate analyzer. Conversely, the fluorescent signals in the other groups were consistent with the negative control group, showing that the *Gl*_crRNA can be utilized with specificity for the identification of *G. lucidum* species (Figure 3D). Second, AGE showed high sensitivity for the identification of *G. lucidum*. It was found that even if the concentration was low to 0.04 ng/μL, AGE can still be successfully tested, and even if the final concentration was increased 5-fold, the fluorescence values did not increase significantly (Figure 3E). Third, in the whole experiment, AGE showed good stability with relatively stable fluorescence values (Figure 3F).

### 3.4 AGE enables identification through one-tube operation

**Figure 4:**
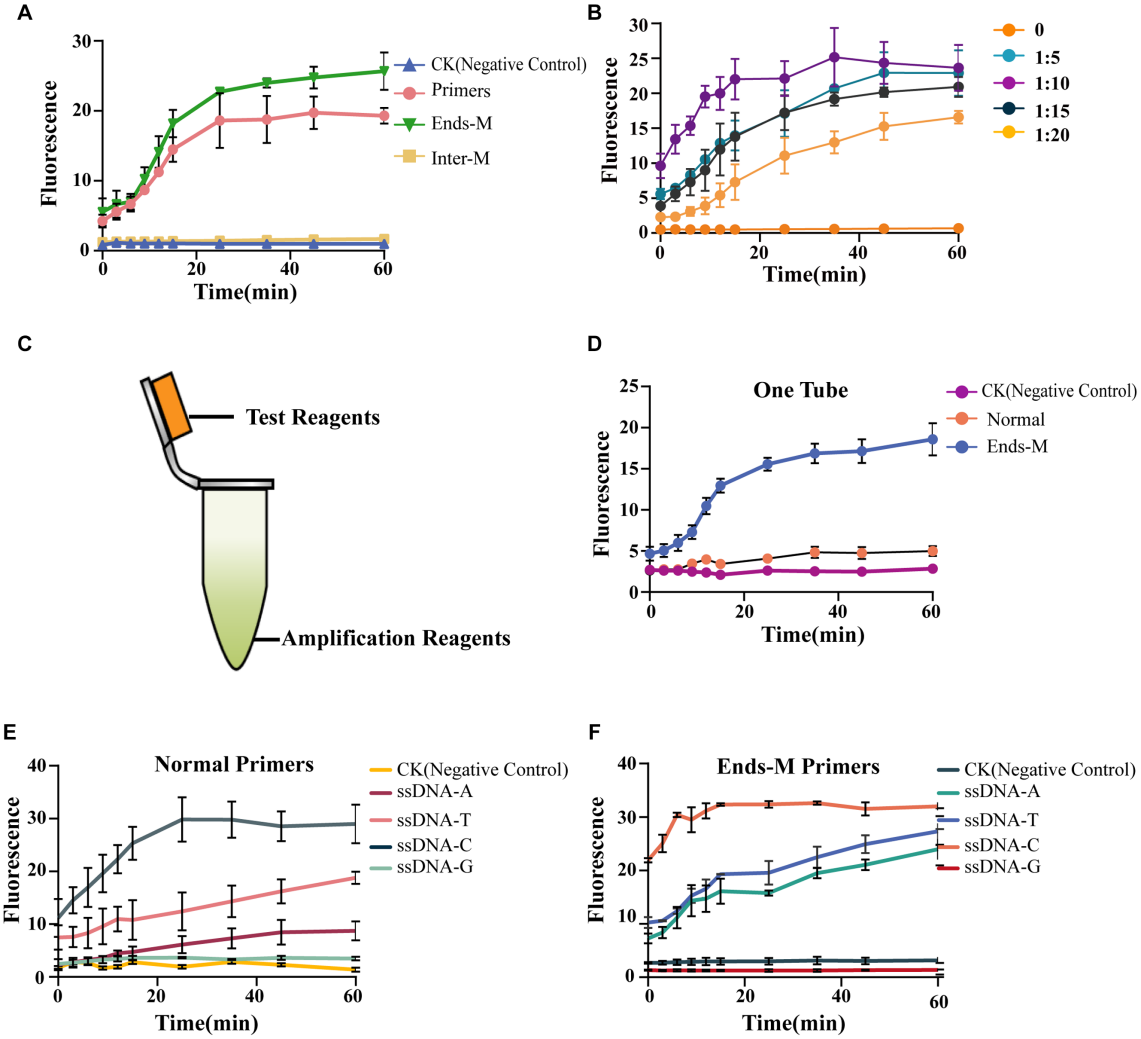
AGE enables identification through one-tube operation. **(A)** The efficiency of AGE using different primers. Each group contained 15-μl ERA product; the CK group contained 15-μl nuclease-free water instead of ERA products. The primer group used normal primers without any modification. The Inter-M group used primers with phosphorothioate modifications in the middle of normal primers. The Ends-M group used the primers with phosphorothioate modifications at both ends of normal primers. **(B)** The test of amplification efficiency. Different groups stand for the volume ratio of the ERA product to the total reaction volume. **(C)** The operation diagram. Test reagents include 2-μl Cas12a, 3.3-μl crRNA, 10-μl NEBuffer 2.1, and 30.7-μl nuclease-free water. Amplification reagents, including ERA kit components. **(D)** Using different primers (normal and Ends-M) to perform AGE. CK group used normal primers without genomic DNA. The normal group used the normal primers and 10-ng/μl genomic DNA. The Ends-M group used phosphorothioate primers with both ends modified and 10-ng/μl genomic DNA. **(E)** The efficiency of different ssDNA reporters with normal primers. CK group contained all reagents except genomic DNA. **(F)** The efficiency of different ssDNA reporters with Ends-M primers. CK group contained all reagents except genomic DNA.

To further reduce the required time and optimize experimental procedures, we tried to substitute the PCR amplification and purification steps in the method with room-temperature amplification. In our study, we used an ERA kit to complete room-temperature amplification without specialized instruments. First, we tested the amplification efficiency with different modified primer pairs (Supplementary Table S2). The results showed that intermediately modified primers (Inter-M) could not amplify *Gl*_target in genomic DNA. It was also determined that the phosphorothioate modification at both ends of the primers (Ends-M) and normal primers was able to complete amplification (Supplementary Figure S4). To verify this, 15 μl of ERA product was added to perform AGE. The findings were consistent with the results above—the Inter-M group showed no fluorescence signal, while other groups exhibited a strong one (Figure 4A). In an initial screening experiment, the intermediately modified primers (Inter-M) were found to be dysfunctional. Further study showed the phosphorothioate modification at both ends of the primers (Ends-M) had better amplification efficiency compared with unmodified primers. Thus, we selected Ends-M primers to explore the optimal volume of ERA product needed to perform AGE, keeping the total volume constant. The results showed that the amplification efficiency was high. Even when the volume ratio of ERA to total reaction volume was as low as 1:20, AGE gave a high fluorescence value (Figure 4B).

Considering the separation of the amplification step from the test step still makes the overall identification process inconvenient, we altered our strategy by performing the amplification at the bottom of a 1.5-ml centrifuge tube and forming the complex on the tube cap (Figure 4C). It was found that only the group of Ends-M primers showed a significant fluorescence signal, while the group with normal primers was nearly identical to the negative control (Figure 4D). To further improve the specificity of our method, different ssDNA reporters were designed to screen for the best fit to the system (Supplementary Table S2). The results showed different ssDNA reporters had significantly dissimilar influences on AGE. The C nucleotide-rich reporters (ssDNA-C) exhibited the strongest fluorescence signal compared with other ssDNA reporters, while other conditions remained constant. When the ssDNA-A reacted with normal primers in a single tube, the observed fluorescence value was identical to the negative control. However, when the ssDNA-A reacted with Ends-M primers, the fluorescence value was significantly greater than the negative control (Figure 4E). Furthermore, the Ends-M group had substantial improvements in sensitivity and a shortened assay time. The fluorescence absorption value of ssDNA-C was nearly three times higher than the group with normal primers at 0 min, and the test time was reduced by half. We found that using Ends-M primers enabled us to improve the sensitivity of AGE and significantly shorten the test time (Figure 4F).

### 3.5 AGE performs well in identifying both Ascomycota and Basidiomycota, especially for closely related species

**Figure 5:**
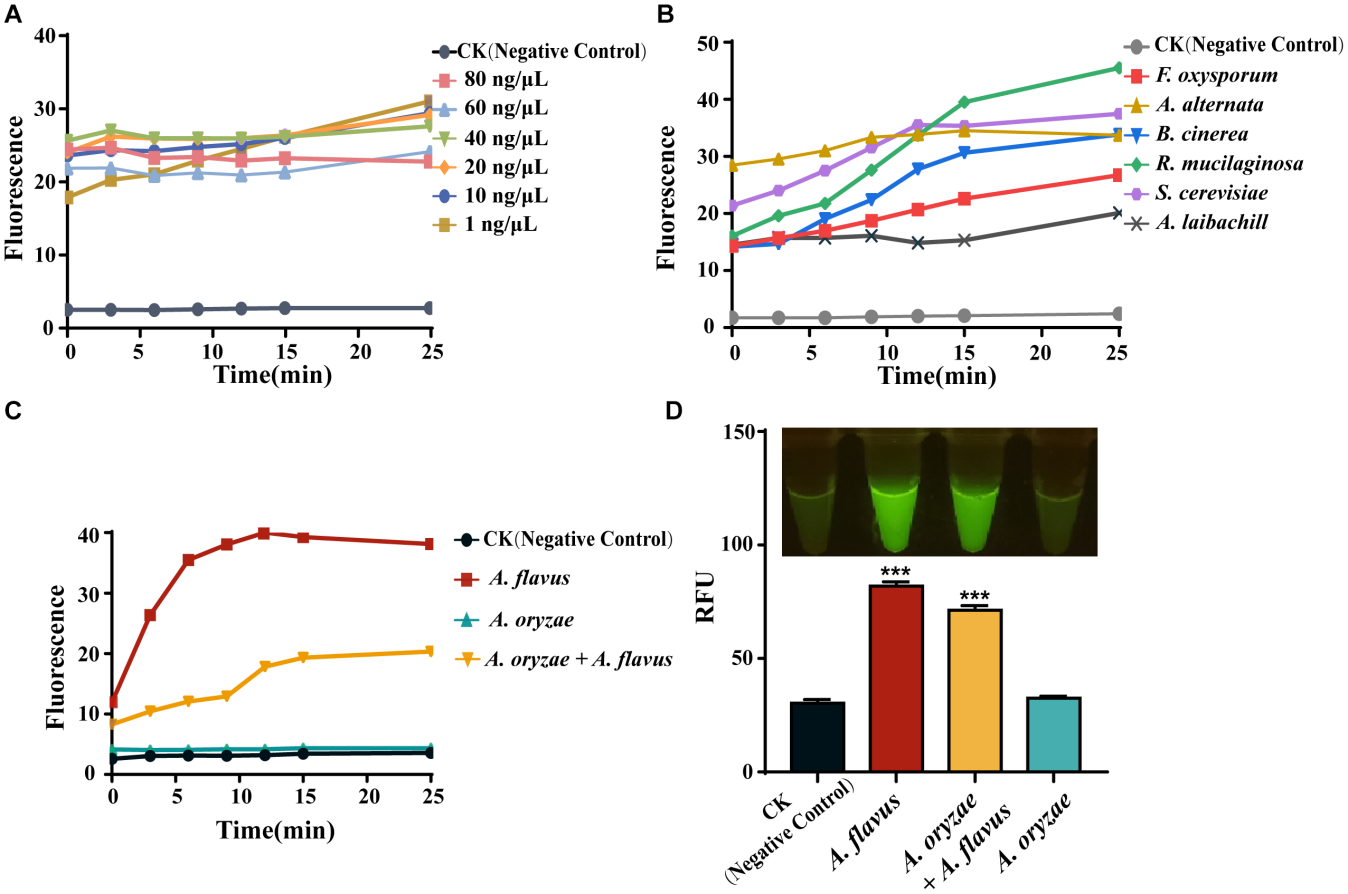
The universality of AGE. **(A)** The sensitivity of the simplified AGE method by measuring fluorescence over time for different starting concentrations of *G. lucidum* genomic DNA. CK group contained all reagents without genomic DNA. **(B)** The identification of different fungi based on the taxonomy. CK group contained all reagents without genomic DNA. **(C)** The identification of closely related species of *A. flavus*. CK group contained all reagents without genomic DNA. **(D)** The identification of closely related species of *A. flavus* by visual fluorescence. CK group contained all reagents without genomic DNA. ***P<0.001.

To apply AGE to fungal identification, we used different concentrations of genomic DNA from *G. lucidum* for identification. The result showed that different concentrations had similar fluorescence absorption values at 25 min, and a stable fluorescence value over time was observed at concentrations equal to or greater than 10 ng/μl (Figure 5A). As 10 ng/μl is the lowest concentration with a stable fluorescence value over time, we recommend 10 ng/μl as the general concentration to perform AGE. Next, AGE was applied to the identification of the other six species based on the above result, which are driven by Ascomycota and Basidiomycota. Further results showed that it can identify all six species with high specificity and sensitivity (Figure 5B).

The *Flavi* section of *Aspergillus* includes both advantageous and detrimental species, such as *A. oryzae*, which is utilized in food fermentation and enzyme production, and *A. flavus*, a food contaminant that produces mycotoxins (Kjærbølling et al., 2020). Therefore, exploring the ability of AGE for closely related species identification would be highly valuable by using it to distinguish *A. flavus* from related species, such as *A. oryzae*. Consistent with the sequencing results, the CRISPR-Cas12a system has successfully achieved identification of *A. flavus* based on specific target sequences (*Af*_target) through fluorescence signal detection. Moreover, we found that this method can also detect the presence of *A. flavus* in *A. oryzae* (Figure 5C). In the visual fluorescence test, this method still exhibits high efficiency, and, more conveniently, we can directly obtain identification results without relying on any equipment (Figure 5D).

### 3.6 Identification of edible fungi and traditional medicines

**Figure 6:**
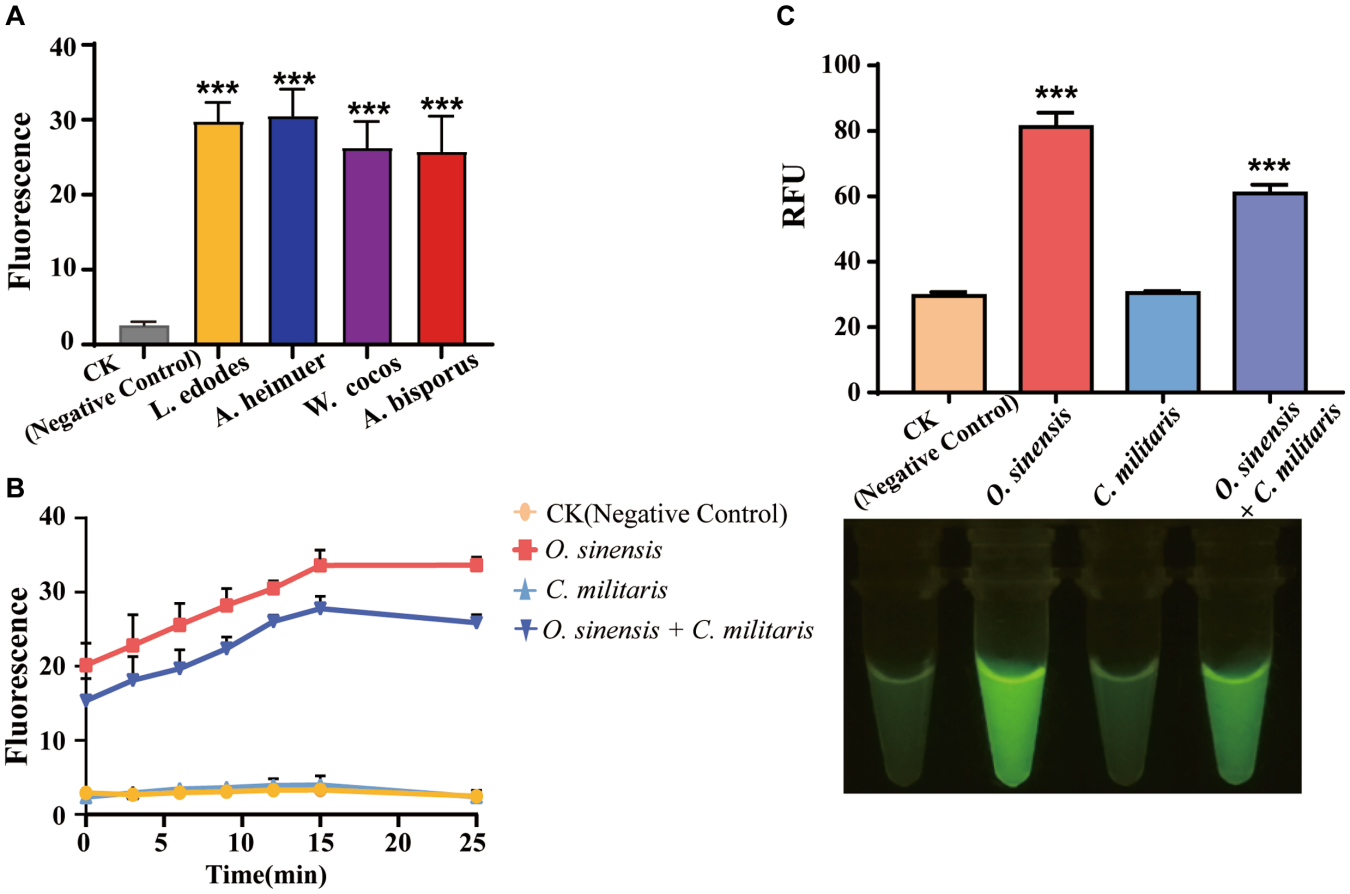
The application of AGE. **(A)** The identification of edible and medicinal fungi. CK group contained all reagents without DNA. **(B)** The identification of adulterant species of *O. sinensis*. CK group contained all reagents without DNA. **(C)** The identification of adulterant species of *O. sinensis* by visual fluorescence. CK group contained all reagents without DNA. ***P<0.001.

Many fungal species are traded in the market for their edible or medicinal value, underscoring the crucial need for accurate and convenient species identification. In our study, we procured three commonly available edible fungal species, namely *L. edodes*, *A. heimuer*, and *A. bisporus*, as well as three medicinal species, namely *W. cocos*, *O. sinensis*, and *G. lucidum*, by random selection from the market. The above results show that AGE can successfully identify *G. lucidum* with several approaches. In addition, other edible and medicinal fungi have also been successfully identified rapidly based on specific Targets, completing the identification process in 30 min with high specificity and sensitivity (Figure 6A).

In this study, particular emphasis was placed on evaluating the discriminatory power of AGE for differentiating between *O. sinensis* and *C. militaris*, especially in the context of detecting the presence of the latter as an adulterant. Specifically, AGE was designed and optimized to target a specific sequence unique to *O. sinensis*, and its ability to accurately distinguish between *O. sinensis* and *C. militaris* was carefully evaluated. The results showed that AGE can achieve the rapid identification of *O. sinensis* from its adulterant species using *Os*_crRNA (Figure 6B). In a laboratory setting, *O. sinensis* can be identified by sequencing amplicon DNA. The successful demonstration of the visual fluorescence method combined with the AGE system addresses the need for on-site fungal identification (Figure 6C).

### 3.7 The workflow of AGE

**Figure 7:**
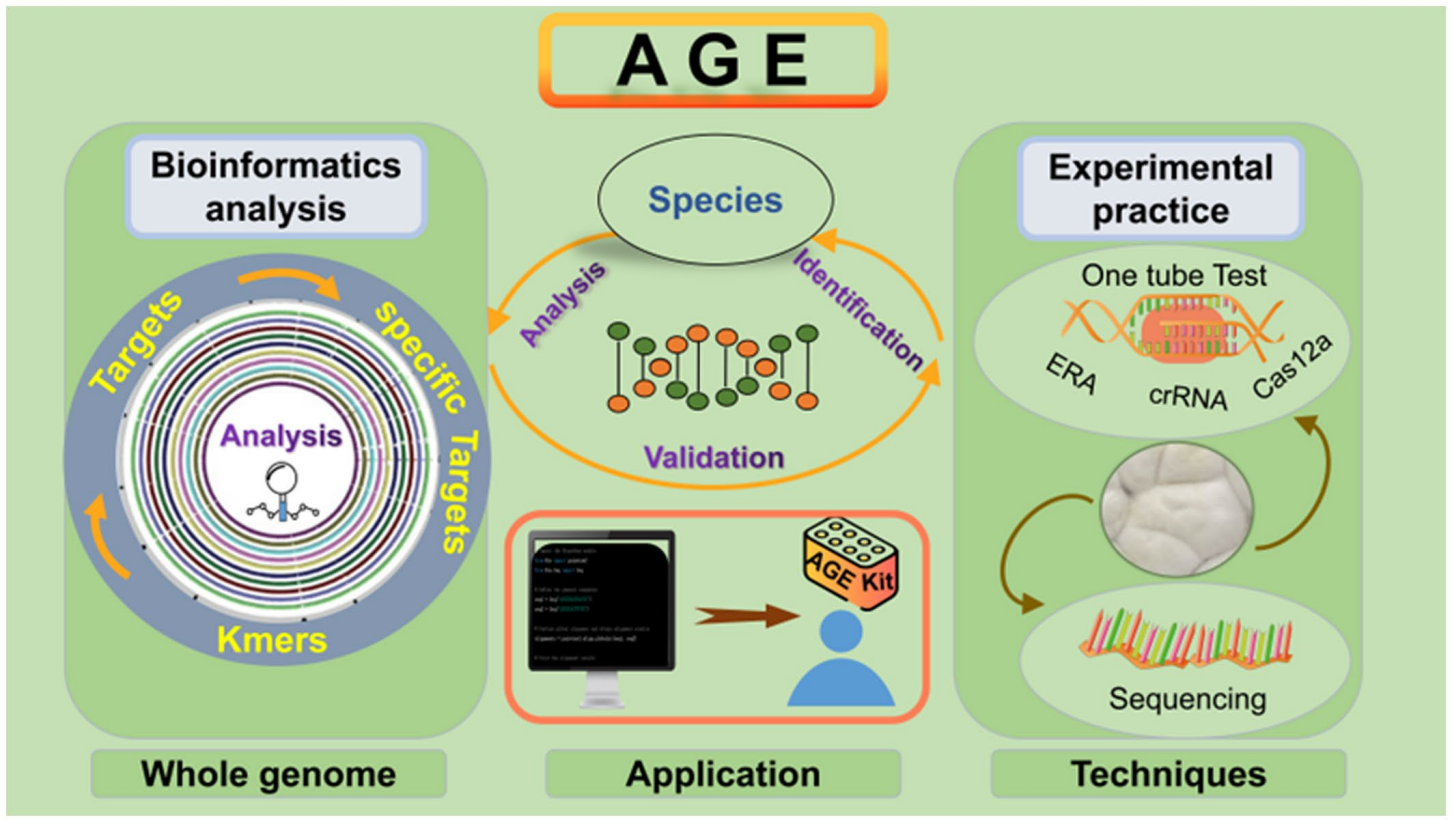
AGE workflow for fungal species identification. To successfully perform AGE, the first step is to cut the whole genome into 23–27 bp and screen the sequences with PAM. The specific whole-genome analysis is the most critical step in forming the Target library, which can be utilized as the standard database for species identification. To identify specific Targets for the indicated species, sequencing and CRISPR-Cas12a system techniques can be implemented. Moreover, the CRISPR-Cas12a system contributes to AGE to realize on-site identification. The genomic DNA of the species is added to the one-tube reaction system. After incubation, the results can be displayed in different ways, including fluorescence intensity measurements and visual fluorescence tests.

Our results demonstrate that AGE involves two steps for identification: bioinformatics analysis and experimental practice. We also provided the workflow of AGE in Figure 7. Through bioinformatic analysis for each species, all 25-bp sequences with PAM were screened. According to the screening principle, sequences with sufficient differences from the genomes of other species were selected to constitute the specific candidate Target library of each individual species. For specific Target recognition, sequencing serves as the gold standard of species identification based on AGE, enabling accurate reading of arbitrary sequences. To achieve laboratory and on-site identification, a crRNA library can easily be constructed by matching each crRNA to its corresponding target sequence. Our experiments have shown that the CRISPR-Cas12a system, together with specific crRNA, serves as a detector, specifically recognizing and binding to the target sequence in the DNA substrate of fungal species while activating Cas12a’s collateral cleavage activity and driving the generation of fluorescence. Conversely, there was no signal with the DNA substrate of related species due to the lack of target sequences.

## 4 Discussion

We have developed a method for the accurate and rapid identification of fungal species based on AGE. First, this study used *Ganoderma lucidum* as the model species and established a species identification method based on whole-genome analysis successfully. Next, we demonstrated that the specific Target of *G. lucidum* can not only be identified through sequencing but also through optimized room-temperature amplification and single-tube CRISPR-Cas12a rapid identification, achieving accurate on-site species identification. Finally, we successfully applied AGE to the identification of a wide range of 13 fungal species, especially for identifying closely related species and detecting traditional medicinal fungi and their adulterants. This capability positions AGE as highly suitable for quality and safety monitoring of food and medicinal materials.

### 4.1 Revolutionizing species identification: AGE’s unprecedented ability to discriminate closely related species, edible and medicinal species at species level

Species-level identification of organisms has been a cornerstone of biology for centuries (Kõljalg et al., 2016). In this study, we identified several fungal species from Ascomycota and Basidiomycota by the specific Targets from the ITS region. Remarkably, specific Targets from other regions of the genome can also be utilized. Given that the current universal barcode for fungal identification is ITS, we prefer specific Targets located in ITS regions that are suitable for the development and validation of the new method. We utilized this strategy to successfully identify several edible and medicinal fungi, including *O. sinensis*, a well-known traditional Chinese medicine (Zhang J. et al., 2020a), and its adulterant *C. militaris* (Liu et al., 2017). More significantly, AGE demonstrates a high level of discriminatory power in distinguishing between *O. sinensis* and *C. militaris*, particularly in detecting the presence of the latter as an adulterant. This capability positions AGE to play a pivotal role in the quality and safety supervision of food, medicinal materials, and health products.

The universal ITS barcode is ineffective in distinguishing closely related species due to its insufficient variability (Schoch et al., 2012; Sun et al., 2022), as evidenced by the identical ITS sequences observed in species *A. flavus* and *A. oryzae* (Supplementary Figure S3). Utilizing the published whole genomes of *A. flavus* (Kjærbølling et al., 2020) and *A. oryzae* (Machida et al., 2005), bioinformatic analysis revealed 83,191 specific Targets in *A. flavus*. Experimental validation of a randomly selected Target successfully distinguished *A. flavus* from its closely related species, *A. oryzae*. Furthermore, the selected *Af*_target has the capability to detect the presence of *A. flavus* in a mixture, rendering it particularly valuable for ensuring food safety and quality control in Chinese medicinal materials. Sequence alignment analysis on *Af*_target amplicons has revealed that this Target is currently unannotated, showing great promise for further research. In the current landscape of fungal species identification, several pressing challenges demand attention. For example, the accurate and timely identification of *Candida albicans* of Ascomycota is needed in medical settings as it is a frequent cause of mucosal and systemic infections (Shokoohi et al., 2021). Agaricomycotina of the division Basidiomycota includes abundant edible fungi, but some species have fatal toxicity (Gressler et al., 2021). In such scenarios, AGE exhibits distinctive advantages in tackling these particular identification challenges. Following the same process outlined in this study would allow for accurate species identification at the species level. Therefore, AGE could address situations where other existing methods may be challenging to employ (Schoch et al., 2012), especially when dealing with closely related species that are highly similar.

### 4.2 AGE: integrating multiple detection approaches for high specificity and sensitivity in species identification

AGE offers a high level of specificity and sensitivity for fungal species identification. As a gold standard for target identification, sequencing enables the capture of any nucleotide variation, enhancing the sensitivity of AGE to single-base differences. Additionally, advancements in sequencing technologies have streamlined the batch verification of large sample numbers (Gao et al., 2023), particularly benefiting laboratories or professional institutions equipped with specialized DNA sequencers and technologists. Moreover, CRISPR-Cas12a contributes to AGE as a simple and on-site species identification method, which is especially important to meet the clinical, customs, and market needs for rapid fungal identification. In the creation of this method, we have integrated amplification, CRISPR-Cas12a, and crRNA into a single tube for streamlined on-site operation. Specifically, we observed that the two sites of phosphorothioate modification on the Ends-M primers are more suitable for amplification in the system of multi-enzyme and complex compounds, which is consistent with published studies (Cai et al., 2018). The modification in the middle sites of primers likely prevents this reaction, which may relate to the structural alterations of the primer pairs. Sensitivity experiments have shown that this system can produce significant fluorescence signals even at low genomic DNA concentrations of 1 ng/μL. Moreover, the accurate identification of different species using a single-tube system confirms that this operation does not compromise the specificity and stability of the AGE. In addition, the C nucleotide-rich reporter exhibited the strongest fluorescence signal while other parameters were kept constant, indicating the highest affinity of this reporter to the crRNA-Cas12a complex. It can rapidly recognize the target DNA of fungal species with minor restrictions, which is consistent with the performance of this system in other organisms like bacteria (Luo et al., 2021) and viruses (Ding et al., 2020). In our study, we have successfully combined a variety of technologies for terminal testing with the CRISPR-Cas12a system, including visual fluorescence and microplate readers, which can address specific needs for different purposes.

### 4.3 The limitations and future of AGE

As with any method, AGE still has some limitations at this stage. First, the availability of fungal genomic sequences is generally considered a major challenge for AGE. Second, public concerns about the requirement for bioinformatics analysis would restrict the widespread adoption and application of this method. Third, while AGE serves as a powerful molecular identification method, it is limited to DNA samples and may encounter challenges when confronted with severe degradation. However, recent research trends refute these concerns. Regarding the availability of genomic data, this will be alleviated by decreasing sequencing costs and the abundance of high-quality fungal genomes (Zhang L. et al., 2020b). A decade ago, there were only approximately 300 fungal genomes published, but today there are over 12,000 genomes publicly available. In 2001, the cost per MB of data was $5,292, and in 2021, it was $0.006. Thus, the easy accessibility of any fungal genome shows that AGE will spearhead the development of genome-based species identification. Moreover, a well-constructed AGE platform could enable anyone to perform species identification, enabling broader use. Advances in bioinformatics enable rapid analysis of high-throughput data. Establishing a comprehensive database with genome analysis steps and qualified crRNA sequences could enhance species identification and aid in identifying fungal pathogens. This database, designed for sustainability with automated updates, could address fungal threats to human, plant, and ecosystem health (Cairns et al., 2016). Thus, AGE does not require extensive bioinformatics skills, as simple software or databases would be available for genomic analysis. Additionally, integrating ultrasensitive and visual detection methods (Ding et al., 2020) into AGE could reduce reliance on specialized instrumentation and shorten testing times. With continued advancements in molecular biology techniques, AGE is poised to achieve instrument-free, visual identification in the foreseeable future. Although AGE cannot be used for the identification of severely degraded samples and non-DNA samples, different chemical detection methods (Wei et al., 2022) can complement this method.

## 5 Conclusion

AGE was established by analyzing and recognizing the species-specific Targets from the whole-genome sequences. We first demonstrated the identification capability of specific Targets in the ITS region. Furthermore, we provided initial evidence for the outstanding identification ability of a specific Target from the genomic “dark matter” for the differentiation of *A. flavus* and *A. oryzae*. Methodological examination showed AGE holds high specificity, sensitivity, and stability, enabling accurate identification of fungal species, including those from the Ascomycota and Basidiomycota phyla. In this study, particular emphasis was placed on evaluating the discriminatory power of AGE for differentiating between *O. sinensis* and *C. militaris*, especially in the context of detecting the presence of the latter as an adulterant. In summary, AGE has been demonstrated to be a rapid, accurate, universal approach for fungal species identification with ultrahigh sensitivity and specificity. In turn, such a simple and robust approach has great potential to drive the development of next-generation genome-based species identification techniques.

## Data availability statement

The datasets presented in this study can be found in online repositories. The names of the repository/repositories and accession number(s) can be found in the article/Supplementary material.

## Author contributions

GQ: Writing – review & editing, Data curation, Formal analysis, Investigation, Methodology, Validation, Visualization, Writing – original draft. LH: Methodology, Visualization, Writing – review & editing. TX: Writing – review & editing, Funding acquisition, Supervision. YG: Validation, Writing – review & editing. QL: Writing – review & editing, Validation. WX: Validation, Writing – review & editing, Methodology, Resources, Software. JS: Writing – review & editing, Conceptualization, Funding acquisition, Project administration.

## Footnotes

^1^https://www.ncbi.nlm.nih.gov

^2^https://www.ncbi.nlm.nih.gov/tools/primer-blast/
