# Supplementary material for: Analysis of Whole-Genome facilitates rapid and precise identification of fungal species
Source: Front Microbiol. 2024 Mar 4;15:1336143. doi: 10.3389/fmicb.2024.1336143 (PMC10946254; doi:10.3389/fmicb.2024.1336143)
Supplement: Supplementary file 1 [file Data_Sheet_1.docx]

**Supplementary** **Information** **for**

**Analysis of Whole-Genome (AGE) Facilitates Rapid and Precise Identification of Fungal Species**

Guihong Qi^a^, Lijun Hao^a^, Yutong Gan^a^, Tianyi Xin^a^, Qian Lou^a^, Wenjie Xu^a^**^*^**, Jingyuan Song^a,b^**^*^**

^a^ Key Lab of Chinese Medicine Resources Conservation, State Administration of Traditional Chinese Medicine of the People’s Republic of China, Institute of Medicinal Plant Development, Chinese Academy of Medical Sciences & Peking Union Medical College, Beijing 100193, China.

^b^ Engineering Research Center of Chinese Medicine Resource, Ministry of Education, Beijing 100193, China.

* Co- Corresponding author Jingyuan Song and Wenjie Xu.

Email: jysong@implad.ac.cn & wjxu@implad.ac.cn

Address: No.151, Malianwa North Road, HaiDian District Beijing 100193

**Supplemental** **method** **and** **result**

**Method**

After amplification of ITS fragments of each species, the purified PCR products were sequenced bidirectionally using Sanger sequencing. Contig assembly and the generation of consensus sequences were performed using Codon Code Aligner. Low-quality sequence data and primer sequences were removed.

PCR amplification of the target was performed in 25 μL reaction mixtures containing 30 ng of genomic DNA, 12.5 μL of 2× Taq PCR MasterMix (Aidlab Biotechnologies Co. Ltd., China), and 1 μL of each forward and reverse primers (2.5 μmol/L). Samples were amplified in an Applied Biosystems VeritiTM Thermal Cycler (Thermo Fisher Co. Ltd., USA), the reaction conditions were as follows: 5 min at 94 °C, followed by 30 cycles of 1 min at 94 °C, 1 min at 50 °C, and 1.5 min + 3 sec/cycle at 72 °C, with a final step of 7 min at 72 °C. The amplified DNA was purified according to the instruction of Qiaquick PCR Purification Kit (Qiagen, Co. Ltd., Germany) and named the product as Gl_target. The amplification results was visualized by 2% (w/v) agarose gel electrophoresis in 1× TAE buffer at 120 V for 30 minutes.

Enzymatic Recombinase Amplification (ERA) for room temperature amplification. The 2-3 μL of genomic DNA was added according to the instruction of the ERA kit (Gendx, Suzhou, China). After amplification, checked the amplification efficiency by 1% (w/v) agarose gel electrophoresis.

**Specificity and cross-reactivity study**

PCR amplification of the Targets was performed in 25 μL reaction mixtures containing 30 ng of genomic DNA, 12.5 μL of 2**×** Taq PCR MasterMix (Aidlab Biotechnologies Co. Ltd., China), and 1 μL of each forward and reverse primers (2.5 μmol/L). Samples were amplified in an Applied Biosystems Veriti^TM^ Thermal Cycler (Thermo Fisher Co. Ltd., USA), the reaction conditions were as follows: 5 min at 94 °C, followed by 30 cycles of 1 min at 94 °C, 1 min at 50 °C, and 1.5 min + 3 sec/cycle at 72 °C, with a final step of 7 min at 72 °C. The amplified DNA was purified according to the instruction of Qiaquick PCR Purification Kit (Qiagen, Co. Ltd., Germany) and the product was recovered, followed by dilution of the purified product to 1ng/ul. Next, prepared the Cas12a-crRNA complex solution. It contained 10 μL NEBuffer 2.1 (10 ×), 2 μL Cas12a (1 μmol/L), 3.3 μL Gl_crRNA (10 μmol/L), and 64 μL nuclease-free water. Next, the solution was incubated at 37 °C for 15 minutes. Upon complex formation, immediately added 10 μL Target (1 ng/μL) from PCR amplification and 4 μL ssDNA-A (10 μmol/L) into it, and subsequently fluorescence intensity was recorded by a microplate reader.

**Result**

The results in Figure S1 show that for species with specific target sequences located in the ITS region, we can only find sequences that are completely identical to the specific target sequences in the corresponding species and not in other species. This indicates that we can use the specific sequences of each species for accurate species identification. At the same time, the results in Figure S2 tell us that *Aspergillus flavus* and *Aspergillus oryzae* do have completely identical ITS sequences, which causes ITS barcoding to fail to distinguish these two closely related species.

To solve this problem, we screened qualified specific target sequences at other genomic locations and designed specific primers for amplification of the target sequences. The results in Figure S3 show that the primers can achieve specific amplification of *A. flavus* and *A. oryzae*, and sequencing results further indicate that this specific sequence only exists in *A. flavus*, while the similar sequence in *A. oryzae* differs from it by 5 nucleotides. Therefore, accurate species identification of *A. flavus* has been achieved.

The result in Figure S4 showed that ERA can achieve room temperature amplification coupled with normal primers or primers with phosphorthioated modifications at both ends.

**The high specificity of each Target.**

Our results show that the Target of each species can only specifically cause a significant fluorescence signal in this species, while there is no obvious fluorescence signal in other species, which indicates the high specificity of the method and does not cause cross-reactivity (Figure S5).


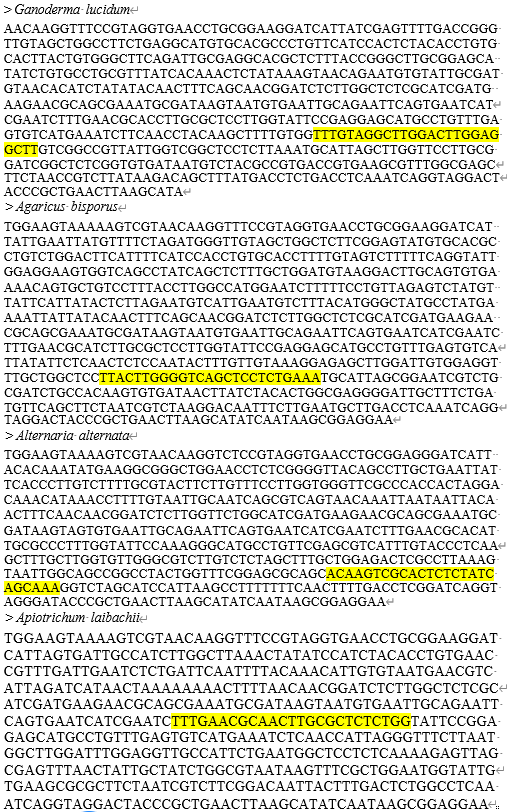
**Fig. S1**


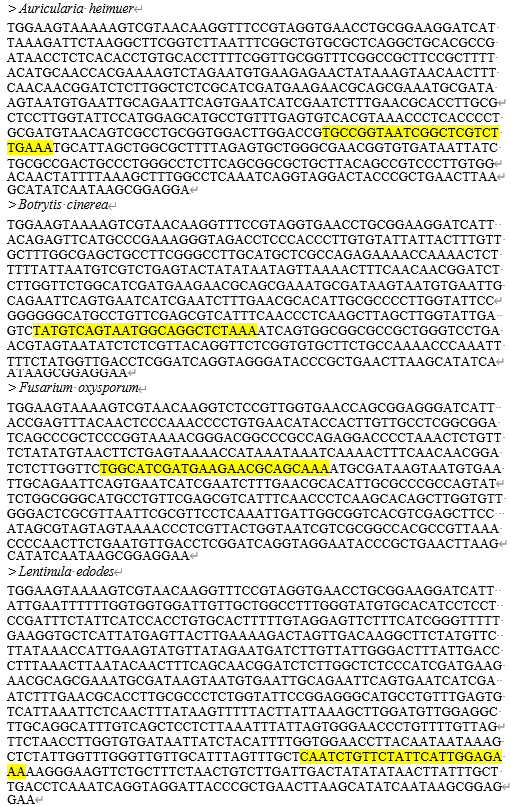


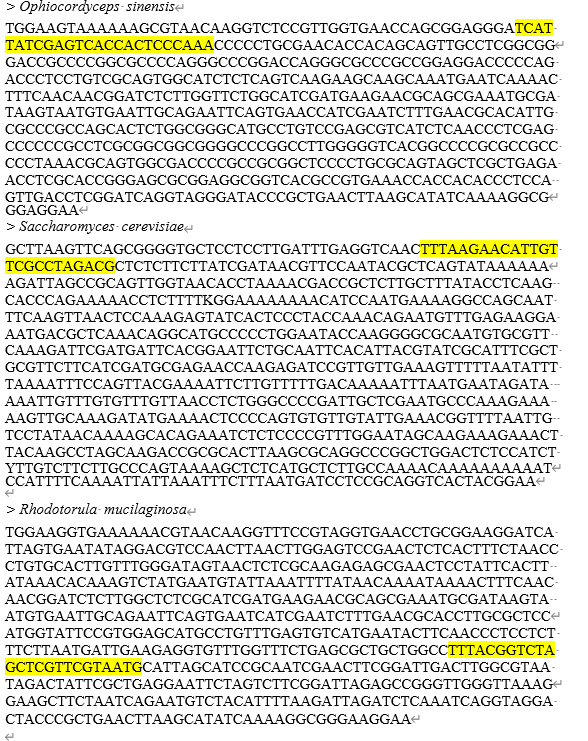


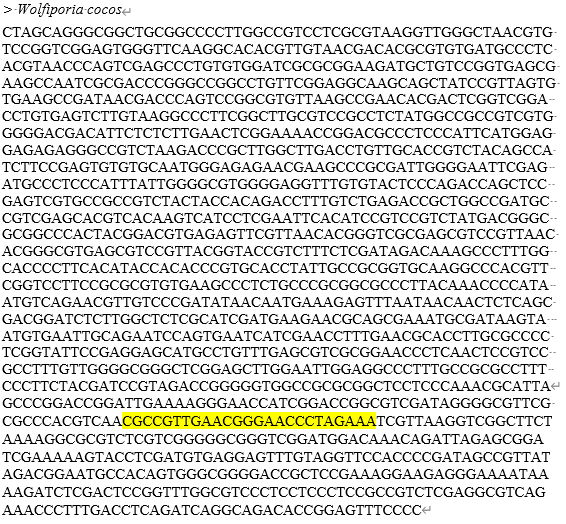
**Figure S1. The sequencing result of specific Targets from ITS region achieved accurate fungal species identification.** The specific target sequence for each species is highlighted in yellow.

**Fig. S2**
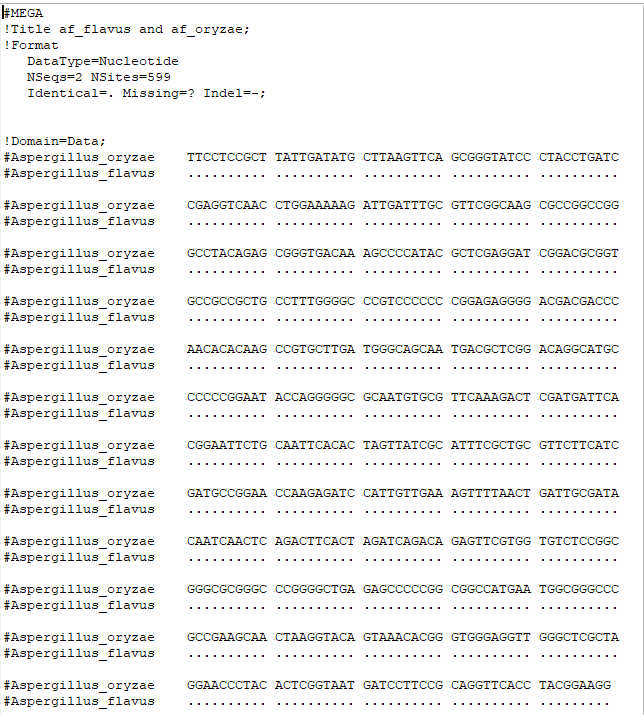


**Figure** **S2.** **The alignment for *A. flavus* ITS sequences with *A. oryzae* ITS sequences.** The dot indicates that the sequence at this specific position in *Aspergillus oryzae* is identical to the corresponding position in *Aspergillus flavus*.


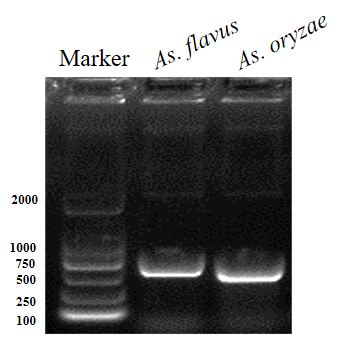
**Fig. S3**

Marker

*A. oryzae*

*A. flavus*

**Figure S3. The** **amplification efficiency of specific primers.** M is the DL2000 DNA marker. *A. flavus* group used the genomic DNA of *Aspergillus flavus*. *A. oryzae* group used the used the genomic DNA of *Aspergillus oryzae*.

**Fig. S4**.


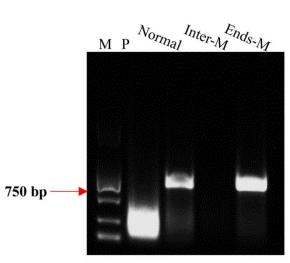


**Figure** **S4.** **ERA amplification efficiency with different primers.** M is the DL2000 DNA marker. P: the positive control of ERA kit; Normal group used normal primers without any modification. Inter-M group used the primers with phosphorthioated modifications in the middle of normal primers. Ends-M group used the primers with phosphorthioated modifications at both ends of normal primers.


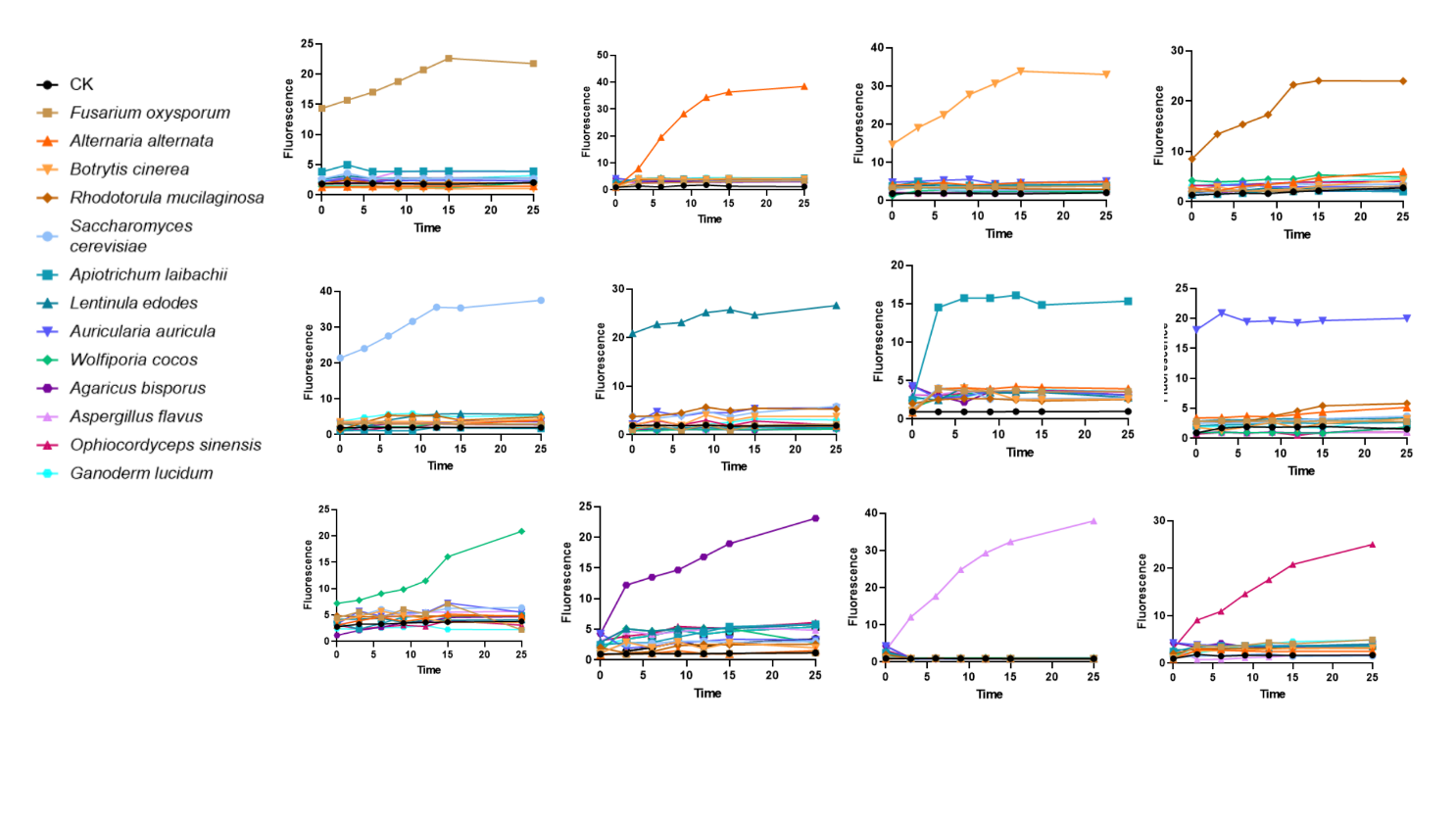
 **Fig. S5**.

**Figure** **S5.** **Specificity and cross-reactivity of species Targets.** The Target of each species can only specifically cause a significant fluorescence signal in this species, while there is no obvious fluorescence signal in other species.

**Table** **S1** **Statistics of Targets in 13 fungal species.**

| Species | Total | Deduplicated | Single copy | Average distance between two potential targets (bp) |
| --- | --- | --- | --- | --- |
| *Agaricus*  *bisporus* | 972,828 | 856,271 | 809,090 | 32 |
| *Alternaria*  *alternata* | 741,076 | 733,539 | 729,877 | 46 |
| *Apiotrichum*  *laibachii* | 341,367 | 338,379 | 337,009 | 90 |
| *Aspergillus*  *flavus* | 1,073,076 | 1,066,016 | 1,052,241 | 35 |
| *Auricularia*  *heimuer* | 834,997 | 710,290 | 660,742 | 60 |
| *Botrytis* *cinerea* | 1,638,675 | 1,604, 191 | 1,590,632 | 26 |
| *Fusarium*  *oxysporum* | 1,354,374 | 1,304,832 | 1,281,940 | 37 |
| *Ganoderma*  *lucidum* | 745,190 | 646,365 | 613, 188 | 64 |
| *Lentinula*  *edodes* | 1,415,488 | 1, 119,783 | 1,054,595 | 32 |
| *Ophiocordyceps.* *sinensis* | 3,432,386 | 1,385,096 | 1, 122, 151 | 33 |
| *Rhodotorula*  *mucilaginosa* | 298,019 | 293,058 | 290,061 | 68 |
| *Saccharomyces* *cerevisiae* | 603,207 | 563,785 | 553,003 | 21 |
| *Wolfiporia*  *cocos* | 851,570 | 856,271 | 809,090 | 59 |

**Table** **S2** Specificity of Targets in ITS region of *Ophiocordyceps.* *sinensis* in *Cordyceps* *militaris*.

| **Targets** **in** **ITS** **region** **of** ***O.***  ***sinensis*** | **Off** **targets** **in** ***C.*** ***militari***  **Chromo** **Posit** **Sequence** **(5’→3’)** **some** **ion** | | |  |
| --- | --- | --- | --- | --- |
| **Name** **Sequence** **(5’→3’)** |  |  |  | **Misma** **tches** |
| \| **Os** **Ta**  **_**  **rget1** \| TTTCACGGCGTGACC GCCTCCGCGC \| \| --- \| --- \| | CP0233 25. 1  CP0233 25. 1 | 1905 TTTCACtttGTGACCGa  549 CgCCGCGC  2018 TTGACGGCcTcAgCG  150 CCggCGCGC | | **5**  **5** |
| \| **Os** **Ta**  **_**  **rget2** \| TTTAGGGGGCGGCGC GGGGCCGTGA \| \| --- \| --- \| | CP0233 28. 1  CP0233 25. 1  CP0233 24. 1 | 1430 TTCcGGGGgGGaGCG  73 GcGCgGTGA  1064 TTTCGGGGGCGatGCc  298 GGGaCGgGA  2535 TTTGGGtGGCaGCtCG  461 GGtCCGTGg | | **5**  **5**  **5** |
| \| **Os** **Ta**  **_**  **rget3** \| TTTCGCTGCGTTCTTC ATCGATGCC \| \| --- \| --- \| | CP0233 22. 1  CP0233 22. 1  CP0233 22. 1  CP0233 22. 1  CP0233 27. 1 | 3682 TTCGCTGCGTTCTTC  721 ATCGATGCC  3704 TTCGCTGCGTTCTTC  264 ATCGATGCC  3693 TTCGCTGCGTTCTTC  491 ATCGATGCC  3715 TTCGCTGCGTTCTTC  030 ATCGATGCC  3548 TTGaCTGCGTTCTgtA  20 TgaATGCC | | **0**  **0**  **0**  **0**  **5** |
| \| **Os** **Ta**  **_**  **rget4** \| TTTGATTCATTTGCTT GCTTCTTGA \| \| --- \| --- \| | CP0233 23. 1  CP0233 24. 1 | 3504 TTGATTCATTgGCTT  636 GCaTCTTtt  6555 TTTGATgCATaTGCTT  396 GgTTCTTGt | | **4**  **4** |
| \| **Os** **Ta**  **_**  **rget5** \| TTTGAACGCACATTG CGCCCGCCAG \| \| --- \| --- \| | CP0233 22. 1  CP0233 22. 1  CP0233 22. 1  CP0233 22 1 | 3682  791  3693  561  3704  334  3715  100 | TTTGAACGCACA TTG CGCCCGCCAG  TTTGAACGCACATTGCGCCCGCCAG  TTTGAACGCACATTGCGCCCGCCAG  TTTGAACGCACATTGCGCCCGCCAG | **0**  **0**  **0**  **0** |

4967 TTTCtACaCgaATTGCG

CP0233 23. 1

CP0233 22. 1

CP0233 22. 1

CP0233 22. 1

CP0233 22. 1

**5**

**Os** **Ta**

**_**

TTTCAACAACGGATC TCTTGGTTCT

**1**

**rget6**

**1**

**1**

**1**

**Os** **Ta**

**_**

**-**

-

**rget7**

**Os** **Ta**

**_**

**5**

**rget8**

789 CCCGCCcG

3682 TTTCAACAACGGATC

696 TCTTGGcTCT

3693 TTTCAACAACGGATC

466 TCTTGGcTCT

3704 TTTCAACAACGGATC

239 TCTTGGcTCT

3715 TTTCAACAACGGATC

005 TCTTGGcTCT

- **-**

TTTGGGAGTGGTGAC TCGATAATGA

TTTGCTTGCTTCTTGA CTGAGAGAT

1351 TTTGCTctCTTCTTGAg

CP0233 24. 1

544 caAGAGAT

**Table S3. All primers and ssDNA reporters used in this study.**

| Oligo name Oligo Sequence (5'→ 3') | | |  |
| --- | --- | --- | --- |
| Primers1 | ITS5F ITS4R | GGAAGTAAAAGTCGTAACAAGG  TCCTCCGCTTATTGATATGC | |
| Inter-M | ITS5F ITS4R | GGAAGTAAA*AGTCGTAACAAGG  TCCTCCGCTTA*TTGATATGC | |
| Ends-M | ITS5F | G*G*AAGTAAAAGTCGTAACAA*G*G | |
|  | ITS4R | T*C*CTCCGCTTATTGATAT*G*C | |
| *Af-*target | F | GCAAGTAGCCCTGAGCATCC | |
|  | R | TACCGTGCAGACCGTGACAC | |
| ssDNA-A |  | 5’-FAM-AAAAAAAAAA-BHQ1-3’ | |
| SsDNA-T |  | 5’-FAM-TTTTTTTTTT-BHQ1-3’ | |
| SsDNA-C |  | 5’-FAM-CCCCCCCCCC-BHQ1-3’ | |
| SsDNA-G |  | 5’-FAM-GGGGGGGGGG-BHQ1-3’ | |

* Stars indicate the locations of phosphorothioate modifications.

1. Nasehi, A. *et* *al.* Leaf Spot on Lettuce (*Lactuca sativa*) Caused by *Stemphylium solani*, a New Disease in Malaysia. *Plant* *disease* **97**, 689, doi:10. 1094/pdis- 10- 12-0902-pdn (2013).

**Table** **S4.** **The** **information** **of** **selected** **species.**

| **Based** **on** **Taxonomy** | | | | |
| --- | --- | --- | --- | --- |
| Phylum | Class | Name | Source | Genome |
| Ascomycota | Dothideomycetes | *Alternaria alternata (Fr.) Keissl* | Strain 3.17853 | GCA 014751505. 1 |
|  | Leotiomycetes | *Botrytis cinerea Pers* | Strain 3.4584 | GCA 000143535.2  _ |
|  | Sordariomycetes | *Fusarium oxysporum Schltdl* | Strain 3.12834 | GCA 013085055. 1  _ |
|  | Saccharomycetes | *Saccharomyces cerevisiae Meyen ex E.C. Hansen* | Strain 2.3849 | GCA 016858175. 1 |
|  | Eurotiomycetes | *Aspergillus flavus Link* | Strain 3. 11958 | GCA_014784225.2 |
|  | Eurotiomycetes | *Aspergillus oryzae Anderson* | Strain 3.7204 | GCA_018140755.1 |
|  | Sordariomycetes | *Ophiocordyceps sinensis (Berk.) G.H. Sung, J.M. Sung, Hywel-Jones & Spatafora******** | Beijing | GCA 002077885. 1  _ |
|  | Sordariomycetes | *Cordyceps militaris (L.) Link******** | Beijing | GCA 008080495. 1 |
| Basidiomycota | Tremellomycetes | *Apiotrichum laibachii (Windisch) A.M. Yurkov & Boekhout* | Strain 2.1972 | GCA 001600735. 1 |
|  | Microbotryomycetes | *Rhodotorula mucilaginosa (A. Jörg.) F.C. Harrison* | Strain 2.1284 | GCA 003055205. 1  _ |
| Medicinal Fungi | Agaricomycetes | *Ganoderma lucidum (Curtis) P. Karst******** | Beijing | GCA 019426095. 1  _ |
|  | Agaricomycetes | *Wolfiporia cocos (Schwein.) Ryvarden & Gilb******** | Huanggang, Hubei | GCA 000344635. 1  _ |
| Edible Fungi | Agaricomycetes | *Agaricus bisporus (J.E. Lange) Imbach******** | Handan, Hebei | GCA 001682475. 1 |
|  | Agaricomycetes | *Lentinula edodes (Berk.) Pegler******** | Huanshang, Hubei | GCA 015476405 1  _ |
|  | Agaricomycetes | *Auricularia heimuer (Klotzsch) Mont******** | Jilin, Jilin | GCA 002287115 1  _ |

***** Stars indicate the medicinal and edible species.
